# Normothermic treatment in acute clinical encephalitis: a case report

**DOI:** 10.1186/1752-1947-2-246

**Published:** 2008-07-25

**Authors:** Mari Terashima, Hiroshi Kataoka, Katsuji Hirai, Satoshi Ueno

**Affiliations:** 1 Department of Neurology, Nara Medical University, Kashihara, Nara 634-8522, Japan; 2 Department of Intensive Care Unit, Nara Medical University, Kashihara, Nara 634-8522, Japan

## Abstract

**Introduction:**

Encephalitis is a common infection of the brain, associated with a high risk of mortality and morbidity despite intensive supportive therapy. This report describes a patient with acute clinical meningoencephalitis who responded dramatically when her body temperature was decreased to normothermia (36 to 37°C) in combination with barbiturate therapy.

**Case presentation:**

A 15-year-old, previously healthy girl presented with a 2-day history of headache and meningeal stiffness and pyrexia. Cranial magnetic resonance imaging showed high-intensity signals in the splenium of the corpus callosum on T2-weighted and diffusion-weighted images. On day 4 of admission, the level of consciousness decreased and ataxic respiration and apnea appeared. After that, fever (body temperature >40°C) developed with remarkable tachycardia. The body temperature was decreased with the use of a forced-air-cooling blanket and head cooling. The core temperature, measured in the bladder, was maintained at between 36 and 37°C for 5 days. During the period of normothermia, thiopental sodium was given continuously for 3 days. After normothermia, the level of consciousness increased without the development of fever, and ventilatory support was withdrawn.

**Conclusion:**

Our experience suggests that normothermic treatment in combination with barbiturate therapy may be an effective option for the management of brain swelling associated with acute meningoencephalitis, particularly when accompanied by a persistent high fever.

## Introduction

Encephalitis is a common infection of the brain, associated with a high risk of mortality and morbidity despite intensive supportive therapy. Hypothermia combined with barbiturate therapy has been used to treat brain swelling and intracranial hypertension [1]. Several investigations have shown that mild hypothermia aimed at reducing body temperature to 34 to 35°C is an effective treatment for acute encephalitis and encephalopathy [2] and has recently been used to treat brain swelling caused by trauma [3]. Mild hypothermia produces fewer complications than deep hypothermia, but can cause conditions such as hypokalemia [2]. On the other hand, using body surface cooling for 24 hours to achieve a core body temperature between 36 and 37°C was reported to be safe in patients with acute stroke [4].

We describe a patient with acute clinical meningoencephalitis who responded dramatically when her body temperature was decreased to normothermia (36 to 37°C) in combination with barbiturate therapy.

## Case presentation

A 15-year-old, previously healthy girl presented with a 2-day history of headache, fever and vomiting. On admission to another hospital, she had meningeal stiffness and pyrexia (body temperature 39°C). Lumbar puncture showed 137 white blood cells (79% lymphocytes)/mm^3^.

**Figure 1:**
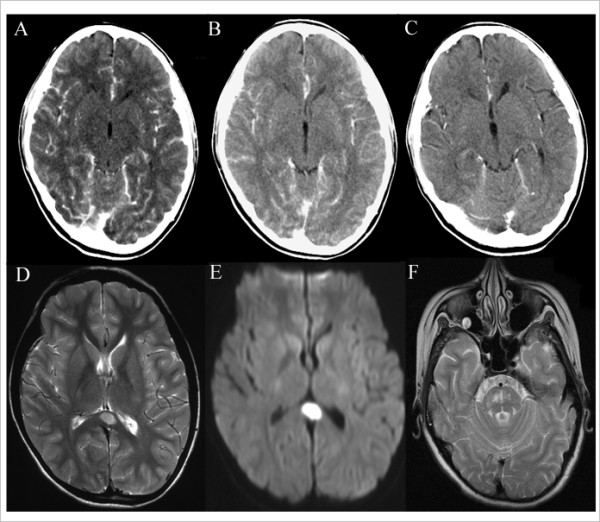
**Cranial computed tomography scans obtained before normothermic treatment and during follow-up**. A cranial computed tomography scan obtained on day 5 (A) before normothermic treatment, showing remarkable meningeal enhancement and brain swelling. Follow-up computed tomography scans obtained on day 12 (B) and day 29 (C), showing reduced meningeal enhancement and brain swelling after normothermic treatment. T2-weighted magnetic resonance imaging (D) and diffusion-weighted magnetic resonance imaging (E) scans, showing increased signal intensity of an ovoid lesion in the splenium of the corpus callosum. A T2-weighted magnetic resonance imaging scan obtained on day 48, showing abnormal increased signals in the pontine (F).

Cranial magnetic resonance imaging (MRI) showed high-intensity signals in the splenium of the corpus callosum (SCC) on T2-weighted and diffusion-weighted images (Figure 1D and 1E). She received intravenous acyclovir and methylprednisolone pulse therapy for a suspected diagnosis of virus encephalitis. However, her consciousness deteriorated and she was transferred to our hospital.

On the day of admission, she presented with disorientation and pyrexia (39.5°C) and could not respond to simple orders. The Glasgow coma score (GCS) was 12; eye opening, verbal response and motor response were 4, 3 and 5, respectively. The heart rate was 118 beats per minute with sinus rhythm. Blood pressure was 120/80 mmHg. Blood cell counts and the results of routine biochemical analysis were normal except for hyponatremia (121 mEq/liter). The osmotic pressure in serum and urine was 277 and 668 mOsm/liter, respectively. Meningeal stiffness was present. The deep tendon reflexes were non-pathological. Lumbar puncture showed 151 white blood cells (89% lymphocytes)/mm^3^, a protein concentration of 78 mg/dl and a glucose concentration of 49 mg/dl, with negative bacterial and tuberculosis cultures. On polymerase chain reaction amplification, herpes simplex virus, varicella-zoster virus, Epstein-Barr virus and cytomegalovirus DNA were all negative in the cerebrospinal fluid (CSF). Infection with various other viruses, such as influenza, parainfluenza, measles and mumps, were excluded by negative serum or CSF antibody titers (or both). Electroencephalography revealed no epileptic discharges. The patient received intravenous acyclovir, dexamethasone and immunoglobulin therapy.

On day 4 after admission, the GCS dropped to 3 (eye opening, verbal response and motor response were 1, 1 and 1, respectively), and ataxic respiration and apnea appeared, leading to respiratory failure requiring ventilatory support. The patient was given intravenous vidarabine and a continuous infusion of propofol, but fever (body temperature >40°C) developed with remarkable tachycardia. The body temperature was decreased with the use of a forced-air-cooling blanket and head cooling. The core temperature, measured in the bladder, was maintained between 36° and 37°C for 5 days. During the period of normothermia, thiopental sodium was given continuously for 3 days. Glycerin and dexamethasone were also given intravenously. After normothermia, the level of consciousness increased and the GCS for eye opening and motor response increased to 4 and 6, respectively, without the development of fever. Verbal response could not be evaluated because the patient had undergone a tracheotomy; however, she could respond to simple orders. Synchronous intermittent mandatory ventilation was decreased from 16 to 8 breaths per minute.

Thirty-six days after admission, ventilatory support was withdrawn. Forty-eight days after admission, cranial MRI showed increased signals in the pontine on T2-weighted images, suggesting osmotic demyelination (Figure 1F), and the high intensity in the SCC had disappeared. At that time, the level of consciousness was normal and the manual muscle test (MMT) scores, based on a 0 to 5 point scale, were 4 and 1 in the upper and lower extremities, respectively. The spinal MRI from the Th3 to L2 level showed no abnormal intensity. Three months after admission, the patient was discharged, with no mental disturbance. The MMT scores were 5 and 1 in the upper and lower extremities, respectively.

Figure 1 shows the serial changes on computed tomography (CT) scans of the brain. A CT scan performed on day 5 (Figure 1A), before normothermia, showed remarkable meningeal enhancement and brain swelling. In contrast, CT scans obtained on day 12, during normothermia (Figure 1B), and on day 29, after normothermia (Figure 1C), showed reduced meningeal enhancement and brain swelling. CSF opening pressure decreased from 160 mm/H_2_O on day 6 (before normothermia) to 130 mm/H_2_O on day 20.

On transcranial Doppler ultrasonography, systolic flow velocities in the right and left middle cerebral arteries before normothermia decreased from 370 to 139 cm per second and from 265 to 140 cm per second, respectively. White blood cells, protein concentrations and interleukin (IL)-6 concentrations in CSF decreased from 101/mm^3^, 74 mg/dl and 69.9 pg/ml on day 6 (before normothermia) to 13/mm^3^, 52 mg/dl and 2.3 pg/ml, respectively, on day 20. The aspartate aminotransferase, alanine aminotransferase and serum sodium concentrations changed from 344 IU/liter, 497 IU/liter and 128 mEq/liter to 159 IU/liter, 320 IU/liter and 132 mEq/liter, respectively, after normothermia. Other laboratory findings were normal after normothermia.

## Discussion

The patient improved clinically without complications after normothermic treatment (36 to 37°C) and showed reduced IL-6 concentrations and leukocyte counts in the CSF.

Conventional hypothermia (body temperature <30°C) has been shown to reduce brain metabolic requirements, which may lessen cerebral edema [5]. Mild hypothermia (34 to 35°C) was also reported to have a marked protective effect against ischemic neuronal injury in experimental models [6], and showed promise for controlling brain swelling [7]. However, these types of hypothermia often cannot maintain the core temperature at the target level for several days and are associated with a risk of complications, such as cardiovascular instability or infection [8]. A previous study demonstrated that a decrease in body temperature of 1 to 3°C can minimize or prevent brain energy failure during hypoxia [8]. Our patient showed a reduction in brain edema on cranial CT after body temperature was decreased by about 3°C. Normothermic treatment may thus minimize or protect against the brain swelling associated with meningoencephalitis.

Several cytokines in serum and CSF are elevated in patients with acute viral encephalitis or encephalopathy [9,10]. IL-6 levels provide particularly valuable information with respect to the diagnosis and severity of encephalitis or encephalopathy [10]. In patients with head injury, moderate hypothermia (32 to 33°C) suppressed increased arterial IL-6 levels, whereas normothermia (36 to 37°C) did not decrease elevated arterial IL-6 levels after brain injury [11]. In our patient, IL-6 levels significantly decreased in response to normothermic treatment plus immunotherapy. This finding suggests that normothermic treatment might suppress the production of cytokines by brain microglia or astrocytes in response to intense inflammation in meningoencephalitis. Moreover, hypothermia has been reported to inhibit the production of IL-6, which may activate neutrophil infiltration [12]. Decreased numbers of leukocytes in the CSF after normothermic treatment also provided evidence that inflammation-induced production of IL-6 was suppressed.

The patient received other treatments, including immunoglobulins, dexamethasone, antiviral agents and anti-edema therapy, which might have affected outcomes (see additional file 1). A previous study showed that corticosteroid treatment was associated with good outcomes in patients with herpes simplex virus encephalitis. Pharmacologically, the good response was ascribed to mechanisms involving the improvement of brain edema and regulation of the host immune response associated with acute encephalitis [13]. In experimental herpes simplex virus encephalitis, dexamethasone treatment suppressed not only the expression of inflammatory genes, but also the expression of viral genes and was associated with neuroprotection and survival [14]. IL-6 secretion in smooth muscle is inhibited by corticosteroids [15].

Recently, the use of intravenous immunoglobulins was associated with relatively good outcomes in autoimmune encephalitis [16]. In addition to normothermia and the effects of barbiturate therapy, immunomodulating, anti-edema or antiviral treatments might have also contributed to the reductions in brain edema or CSF cytokine levels in our patient.

The patient had severe hyponatremia and MRI scans showed symmetric hyperintensity in the pons, confirming the diagnosis of osmotic demyelination syndrome [17]. Severe hyponatremia may be caused by a variety of mechanisms, including hypovolemia, cerebral salt wasting syndrome or inappropriate secretion of antidiuretic hormone [18]. Although direct evidence is lacking, the hyponatremia in our patient might have been caused by hypovolemia due to the persistent high fever or to inappropriate secretion of antidiuretic hormone as the osmotic pressure of serum was less than that of urine. The total daily correction in our patient was less than 10 mmol/liter/day [17], but the patient presented with paraplegia as a residual symptom. Among 34 patients with osmotic demyelination syndrome that were followed up, 11 had a complete recovery but 10 patients had some persistent deficits, similar to our patient [19]. However, the paraplegia in our patient was not consistent with only osmotic demyelination at the pons. Although the cause of paraplegia was unclear on spinal MRI, diseases other than osmotic demyelination syndrome were suspected.

MRI showed transient high-intensity signals in the SCC, which have rarely been demonstrated clinically in encephalitis or encephalopathy [20]. Previous reports have introduced a concept termed 'intramyelinic edema', a non-degenerative change characterized by pathological and neuro-imaging findings of Canavan disease or maple syrup urine disease, associated with water collection between the myelinic lamellae and decreased apparent diffusion coefficient values [21]. A recent pathophysiologic study examining various neuro-imaging findings with techniques such as diffusion tensor MRI or magnetic resonance spectroscopy demonstrated intramyelinic (intercellular) edema [22]. Although we did not perform similar neuro-imaging studies, the reversibility of SCC lesions on diffusion-weighted imaging in our patient may also support the presence of intramyelinic (intercellular) edema. However, confirmation of an association between acute meningoencephalitis and SCC lesions must await further studies.

Although the patient had resultant paraplegia, normothermic treatment in combination with barbiturate therapy plus immunotherapy prevented a lethal outcome directly caused by acute encephalitis.

## Conclusion

Our experience suggests that normothermic treatment in combination with barbiturate therapy may be an effective option for the management of brain swelling associated with acute meningoencephalitis, particularly when accompanied by a persistent high fever.

## Abbreviations

CSF: cerebrospinal fluid; CT: computed tomography; GCS: Glasgow coma score; IL: interleukin; MMT: manual muscle test; MRI: magnetic resonance imaging; SCC: splenium of the corpus callosum.

## Competing interests

The authors declare that they have no competing interests.

## Authors' contributions

MT, HK and KH reviewed the existing literature and drafted the manuscript, which was edited by HK and SU. HK reviewed and selected radiology images. All authors read and approved the final manuscript.

## Consent

Written informed consent was obtained from the patient's next-of-kin for publication of this case report and any accompanying images. A copy of the written consent is available for review by the Editor-in-Chief of this journal.

## Supplementary Material

Additional file 1 Course. Symptoms and treatment during hospitalization period. Click here for file
