# Supplementary figures and images for: Normothermic treatment in acute clinical encephalitis: a case report
Source: J Med Case Rep. 2008 Jul 25;2:246. doi: 10.1186/1752-1947-2-246 (PMC2500027; doi:10.1186/1752-1947-2-246)

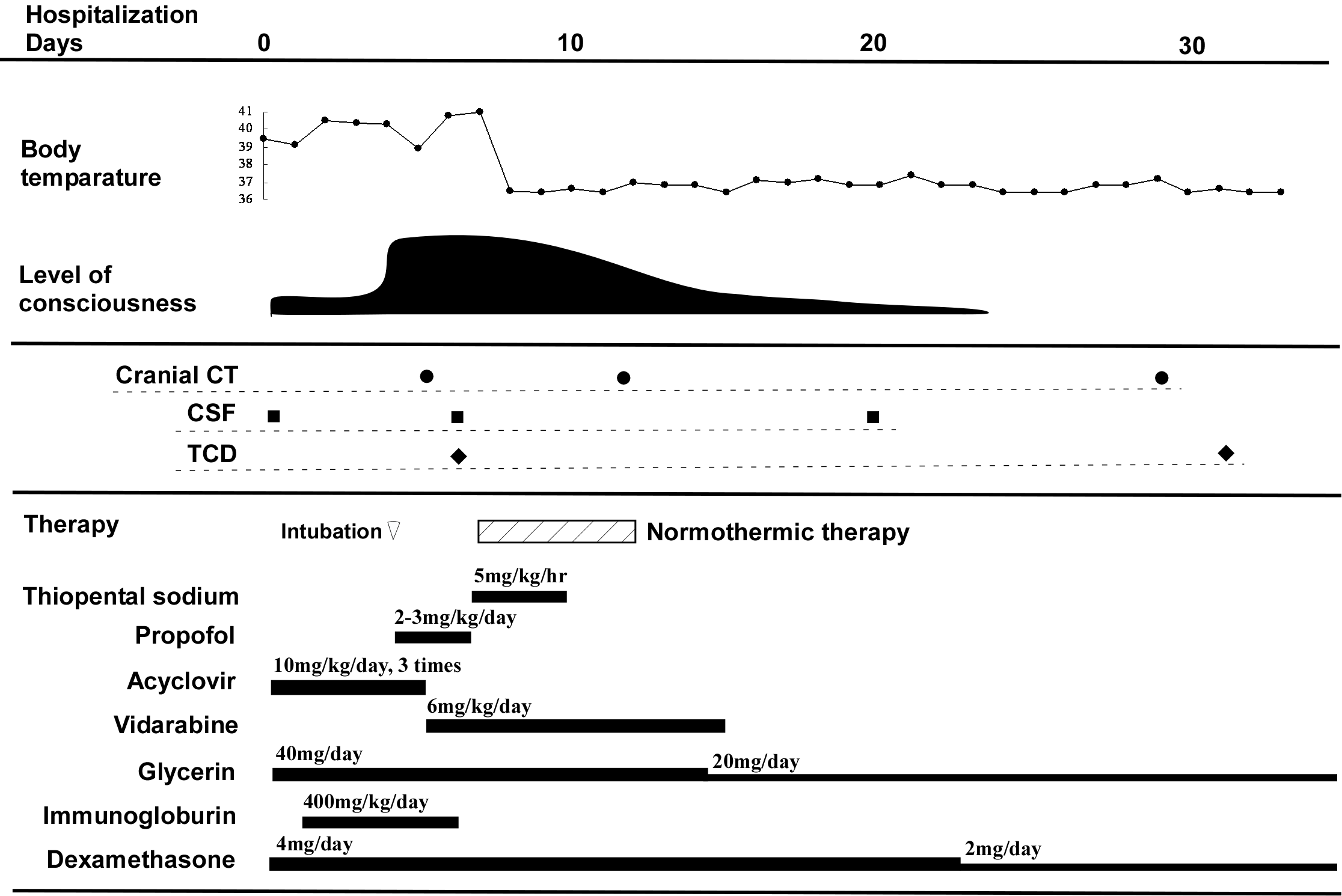

Supplement: Additional file 1 — Course. Symptoms and treatment during hospitalization period. [file 1752-1947-2-246-S1.tiff]
